# Agonistic and Antagonistic Interactions between Chlorhexidine and Other Endodontic Agents: A Critical Review

**Published:** 2014-12-24

**Authors:** Zahed Mohammadi, Luciano Giardino, Flavio Palazzi, Saeed Asgary

**Affiliations:** a * Iranian Center for Endodontic Research, Research Institute of Dental Sciences, Shahid Beheshti University of Medical Sciences, Tehran, Iran; *; b * Iranian National Elites Foundation, Tehran, Iran; *; c * Department of Periodontology, Endodontology, Pharmacology and Microbiology, Dental School, University of Brescia, Italy; *; d * Department of Oral and Maxillofacial Science, University of Naples, Italy*

**Keywords:** Calcium Hydroxide, Chlorhexidine, Ethylenediaminetetraacetic Acid, Interaction, Mineral Trioxide Aggregate, Sodium Hypochlorite

## Abstract

Root canal irrigants play a significant role in elimination of the microorganisms, tissue remnants, and removal of the debris and smear layer. No single solution is able to fulfill all these actions completely; therefore, a combination of irrigants may be required. The aim of this investigation was to review the agonistic and antagonistic interactions between chlorhexidine (CHX) and other irrigants and medicaments. An English-limited Medline search was performed for articles published from 2002 to 2014. The searched keywords included: chlorhexidine AND sodium hypochlorite/ethylenediaminetetraacetic acid/calcium hydroxide/mineral trioxide aggregate. Subsequently, a hand search was carried out on the references of result articles to find more matching papers. Findings showed that the combination of CHX and sodium hypochlorite (NaOCl) causes color changes and the formation of a neutral and insoluble precipitate; CHX forms a salt with ethylenediaminetetraacetic acid (EDTA). In addition, it has been demonstrated that the alkalinity of calcium hydroxide (CH) remained unchanged after mixing with CHX. Furthermore, mixing CHX with CH may enhance its antimicrobial activity; also mixing mineral trioxide aggregate (MTA) powder with CHX increases its antimicrobial activity but this may negatively affect its mechanical properties.

## Introduction

Chlorhexidine (CHX) is a synthetic cationic bisguanide consisting of two symmetric 4-cholorophenyl rings and two biguanide groups, connected by a central hexamethylene chain [1]. It is used in dentistry because of being effective against both gram-positive and gram-negative bacteria as well as yeasts (mycobacteria and bacterial spores are resistant to CHX) and its substantivity and relatively low toxicity [2]. CHX is a positively charged hydrophobic and lipophilic molecule that interacts with phospholipids and lipopolysaccharides on the cell membrane of bacteria and then enters the cell through some type of active or passive transport mechanism [3]. Its efficacy is due to the interaction of the positively charged molecule with the phosphate groups on microbial cell walls with negative charge thereby altering the cells’ osmotic equilibrium [4, 5]. Activity of CHX is pH dependent and is greatly reduced in the presence of organic matter.

This increases the permeability of the cell wall, which allows the CHX molecule to penetrate into bacteria. CHX is a base and is stable in the salt form. The most common oral preparation, CHX gluconate, is water-soluble and at physiologic pH level it readily dissociates and releases the positively charged CHX component [1]. At low concentrations of CHX (0.2%), lower molecular weight substances such as potassium and phosphorous will leak out of the cell. On the other hand, at higher concentrations (2%), CHX is bactericidal and precipitation of the cytoplasmic contents results in cell death [4].

Different endodontic agents used as intracanal medicaments or irrigants may interact with CHX. For instance, interaction of sodium hypochlorite (NaOCl) mixed with chlorhexidine (CHX) produces a brown precipitate containing para-chloroaniline (PCA) which is not only toxic but also interferes with canal sealing [2]. Regarding intracanal calcium hydroxide (CH) medication, it is proved that mixing CH with CHX increases its antibacterial effects [6].

Exposure to CHX decreases the push-out bond strength of mineral trioxide aggregate (MTA) to dentin [7]. However, the antibacterial effect of MTA elevated after mixing with CHX [8, 9]. Moreover, mixing MTA with CHX did not alter the sealing ability of MTA [10].

Chelating agents such as ethylenediaminetetraacetic acid (EDTA) can interact with CHX, as it is shown that subsequent use of CHX, NaOCl and EDTA can cause a color change in dentine [10].

The aim of the present critical review is to determine the interaction between CHX and different endodontic agents according to the results of previous studies published from 2002 to 2014.

## Materials and Methods

***Retrieval of literature***

An English-limited Medline search was performed through the articles published from 2002 to 2014. The searched keywords included “chlorhexidine AND sodium hypochlorite (NaOCl)”, “chlorhexidine AND ethylenediaminetetraacetic acid (EDTA)”, “chlorhexidine AND calcium hydroxide (CH)”, and “chlorhexidine AND mineral trioxide aggregate (MTA)". Then, a hand search was done in the references of collected articles to find more matching papers.

## Results

A total of 1095 articles were found which in order of their related keywords are “567-chlorhexidine AND NaOCl”, “255-chlorhexidine AND EDTA”, “252-chlorhexidine AND CH” and “21-chlorhexidine AND MTA".

***Combination of irrigants and medicaments with CHX***

Despite having important useful properties, CHX possesses some drawbacks as well. One of its important drawbacks is lacking tissue solubility, which is one of the most important properties of a standard irrigation solution [11]. Therefore, CHX cannot be used as a routine root canal irrigant and should be used as final rinse following root canal irrigation with NaOCl [11].

A suggested clinical protocol by Zehnder [12] for dentin treatment before root canal filling consists of: *a*) irrigation with NaOCl to dissolve the organic components, *b*) irrigation with EDTA to assist in the elimination of the smear layer and finally, *c*) irrigation with CHX to increase the anti-microbial spectrum of activity and impart substantivity.

Studies have demonstrated that in retreatment cases where microbial species like *Enterococcus faecalis* (*E. faecalis*) and *Candida albicans* (*C. albicans*) are the main causes of treatment failure, CH is ineffective against these two species [13]. Therefore, combination of CHX with CH is considered favorable and for this reason the effects of this combination on the properties of each of the mentioned materials needs to be evaluated. Moreover, considering the drawbacks of CHX, combining it with other irrigants and medicaments seems to be important for improving its action as an endodontic irrigant.

***Interaction between CHX and NaOCl***

The combined use of NaOCl and CHX has been advocated to enhance their antimicrobial properties. In other words, a final rinse with CHX offers the advantage of substantivity (due to its affinity to dentine hydroxyl apatite) which prolongs the antimicrobial activity of CHX [14]. However, the disadvantage is that the when NaOCl is mixed with CHX, an orange-brown particle (PCA) is formed which results in precipitation a chemical smear layer that covers the dentinal tubules thus interfering with the seal of the root filling [15]. In addition, this precipitate changes the color of the tooth and is cytotoxic [2, 15].

A study used electrospray ionization quadrupole time-of-flight mass spectrometry (ESI-QTOF-MS) analyses to investigate the byproducts formed after combination of CHX and NaOCl [16]. Findings revealed that 2% CHX gel and solution immediately produced PCA precipitate when combined with 1, 2.5 and 5.25% NaOCl solutions, but in combination with 0.16% NaOCl an orange-white precipitate was formed [16].

It seem that the oxidizing activity of NaOCl causes chlorination of the guanidino nitrogens of the CHX [17]. Basrani *et al.* [17] detected the presence of PCA in this orange-brown precipitate. On the other hand, several other studies which used different methodologies failed to detect it [16, 18, 19]. PCA is mutagenic and cytotoxic in microorganisms [14]. Some concerns over possible carcinogenicity of PCA has also been expressed [14]. Basrani *et al.* [17] tested concentrations ranging from 0.023 to 6% of NaOCl to investigate the minimum NaOCl concentration required to form a precipitate when mixed with 2% CHX. Results showed that instant color change occurred in all samples from dark brown to light orange. Precipitation was induced with 0.19% NaOCl with varying amounts of material formed in the different mixtures [17].

Studies have been undertaken to elucidate the chemical composition of the flocculate produced by the association of NaOCl with CHX [16-21]. Marchesan *et al.* [20] combined different concentrations of NaOCl (0.5, 2.5, and 5%) and CHX (0.2-2%) with variable proportions which resulted in immediate formation of a brownish flocculate. Using X-ray photoelectron spectroscopy (XPS) and time-of-flight secondary ion mass spectrometry (ToF-SIMS), Basrani *et al.* [17], showed that PCA was present at concentrations directly related to the NaOCl concentration.

Krishnamurthy and Sudhakaran [22] detected PCA following mixing 2.5% NaOCl with 2% CHX. Chhabra *et al.* [23] showed that PCA is toxic and carcinogenic. Also by using environmental scanning electron microscopy (SEM), the influence of irrigation on debris removal and patency of dentinal tubules was assessed [24]. Findings revealed that there was no difference in remaining debris; however there was a reduction in number of patent dentinal tubules in the coronal and middle thirds when irrigated with 5.25% NaOCl or when combined with 2% CHX [24]. Using SEM, Valera *et al.* [25] assessed the percentage of patent and occluded tubules after root canal instrumentation and irrigation with 2.5% NaOCl combined with 2% CHX in liquid or gel forms, intercalated by physiologic saline, with half of the experimental groups receiving a final flush with 17% EDTA. Findings indicated that 2% CHX gel caused the highest number of open dentinal tubules, whereas 2% CHX liquid presented the lowest. The additional using of EDTA and physiologic saline as a final flush improved the cleaning and debris removal. Akisue *et al.* [26] compared the effects of combining 1% NaOCl and 2% CHX on dentinal permeability using rhodamine dye leakage, and found that the mixture of NaOCl and CHX caused a reduction of permeability only in the apical third. Vivacqua-Gomes *et al.* [27] showed that a precipitate formed after combining 1% NaOCl and 2% CHX gel, which stained the dentin and adhered to the canal walls. They suggested that this precipitate enhanced dye penetration in obturated teeth [27].

In summary, the combination of NaOCl and CHX causes color changes and the formation of a neutral and insoluble precipitate, which may interfere with the seal of the root filling. Therefore, drying the canal with paper points before the final CHX rinse is suggested.

***Interaction between CHX and chelators***

In an *in vitro* study on bovine dentin slices using atomic absorption spectrophotometry, Gonzalez-Lopez *et al.* [28] assessed the effect of adding 1% CHX and 10 to 20% citric acid (CA) on the demineralizing capacity of CA. Results showed that after 3, 10, and 15 min of immersion, the decalcifying effect of CA remained unchanged [28].

Akisue *et al.* [26] showed that using 15% CA followed by 2% CHX caused the formation of a milky solution with no precipitation. Gonzalez-Lopez *et al.* [28] and Rasimick *et al.* [29] demonstrated that it was difficult to obtain a homogeneous solution when mixing CHX with EDTA and a precipitate composed chiefly of the original components formed. Gonzalez-Lopez *et al.* [28] showed that it is not possible to obtain a homogenous solution by mixing 17% EDTA and 1% CHX. Prado *et al.* [16] showed that after combination with EDTA, CHX produced a white-milky precipitate, related to the acid-base reactions. When combined with saline and ethanol, a salt precipitation was noticed. Furthermore, no precipitate was observed when CHX was used together with distilled water, CA or phosphoric acid [16].

In summary, CHX forms a salt with EDTA rather than undergoing a chemical reaction.

***Interaction between CHX and CH***

The optimal antimicrobial activity of CHX is achieved within a pH range of 5.5 to 7.0 [13]. Therefore, it seems that alkalinizing the pH by adding CH to CHX, precipitates CHX molecules and decreases its effectiveness. However, it has been demonstrated that the alkalinity of CH in the mixture remained unchanged. Therefore, the usefulness of mixing CH with CHX still remains unclear and controversial [30].

When used as an intracanal medicament, CHX was more effective than CH in eliminating *E. faecalis* harbored within the dentinal tubules [13, 30]. In a study by Almyroudi *et al.* [31], all of the tested CHX formulations including a 50:50 mixture of CHX and CH, were efficient in eliminating *E. faecalis* from the dentinal tubules; a 1% gel CHX worked slightly better than the other preparations. These findings were corroborated by Gomes *et al.* [4] in bovine dentine and Schafer and Bossmann [32] in human dentine where 2% gel CHX had greater activity against E*. faecalis*, followed by liquid CHX and CH and then CH used alone. Using agar diffusion test, Haenni *et al.* [33] could not demonstrate any additive antibacterial effect by mixing CH powder with 0.5% CHX. In fact, they showed that CHX had reduced antibacterial action. However, CH did not lose its antibacterial properties in such a mixture [33]. This may be due to the deprotonation of CHX at pH levels greater than 10, which reduces its solubility and alters its interaction with bacterial surfaces as a result of altered charge of the molecule [33]. Ercan *et al.* [34] showed that 2% gel CHX was the most effective agent against *E. faecalis* inside human dentinal tubules, followed by a CH mixed with 2% CHX, whilst CH alone was totally ineffective, even after 30 days. The 2% gel CHX was also significantly more effective than the CH and 2% CHX mixture against *C. albicans* at seven days, although there was no significant difference at 15 and 30 days. CH alone was completely ineffective against *C. albicans*. In another study on primary teeth, 1% CHX gluconate gel with and without CH, was more effective against *E. faecalis* than CH alone within a 48-h period      [35].

Schafer and Bossmann [32] reported that 2% CHX gluconate was significantly more effective against *E. faecalis* than CH used alone, or a mixture of the two. This was also confirmed by Lin *et al.* [36]. In a study by Evans *et al.* [37] using bovine dentine, 2% CHX with CH was shown to be more effective than CH mixed with water. In an animal study, Lindskog *et al. *[38] reported that teeth dressed with CHX for 4 weeks had reduced inflammatory reactions in the periodontium (both apically and marginally) and less root resorption. Waltimo *et al.* [39] reported that 0.5% CHX acetate was more effective in killing *C. albicans* than saturated CH, while CH combined with CHX was more effective than CH used alone.

In summary, combined use of CHX and CH in the root canal may generate excessive reactive oxygen species, which may potentially kill various root canal pathogens. In addition, it has been demonstrated that the alkalinity of CH when mixed with CHX remained unchanged. Furthermore, mixing CHX with CH may enhance its antimicrobial activity.

***Interaction between CHX and MTA***

MTA is marketed in gray and white colored preparations: both contain 75% Portland cement, 20% bismuth oxide and 5% gypsum by weight. MTA is a hydrophilic powder which requires moisture for setting. Traditionally, MTA powder is mixed with supplied sterile water in a 3:1 powder/liquid ratio. Different liquids have been suggested for mixing with the MTA powder such as lidocaine anesthetic solution, NaOCl and CHX [40].

Stowe *et al.* [41] determined the effect of substituting sterile water with 0.12% CHX as MTA liquid on the antimicrobial activity of white MTA. They found that 0.12% CHX enhanced the antimicrobial activity of MTA. This finding was confirmed by Holt *et al.* [42]. Hernandez *et al.* [43] compared the percentage of apoptotic cells and the cell cycle profile of fibroblasts and macrophages exposed to either MTA mixed with CHX or MTA mixed with sterile water. Results showed that MTA specimens containing CHX induced apoptosis of macrophages and fibroblasts. In contrast, no change in the proportion of apoptotic cells was observed when sterile water was used to prepare the specimens.

Cell cycle analysis showed that exposure to MTA/CHX decreased the percentage of fibroblasts and macrophages in S phase (DNA synthesis) as compared with exposure to MTA/water. On the other hand, Sumer *et al.* [44] examined the biocompatibility of MTA mixed with CHX histopathologically. They found that MTA/CHX was encapsulated by fibrous connective tissue, which indicates that it was well tolerated by the tissues. Yan *et al.* [45] found that CHX had no negative effect on the bond strengths of MTA-dentin *in vitro*. Kogan *et al.* [46] found that MTA paste prepared with CHX did not set. Furthermore, Holt *et al.* [42] found that MTA mixed with sterile water always had higher compressive strengths than MTA mixed with CHX. Shahi *et al.* [47] evaluated the sealing ability of white and gray MTA mixed with distilled water and 0.12% CHX when used as retrograde filling material. Results showed that CHX had no negative effect on the sealing ability of MTA.

Overall, it can be concluded that mixing MTA powder with CHX increases its antimicrobial activity but may have a negative effect on its mechanical properties.

## References

[1] Greenstein G, Berman C, Jaffin R (1986). Chlorhexidine An adjunct to periodontal therapy. J Periodontol.

[2] Kolosowski KP, Sodhi RN, Kishen A, Basrani BR (2014). Qualitative Analysis of Precipitate Formation on the Surface and in the Tubules of Dentin Irrigated with Sodium Hypochlorite and a Final Rinse of Chlorhexidine or QMiX. J Endod.

[3] Athanassiadis B, Abbott PV, Walsh LJ (2007). The use of calcium hydroxide, antibiotics and biocides as antimicrobial medicaments in endodontics. Aust Dent J.

[4] Gomes BP, Souza SF, Ferraz CC, Teixeira FB, Zaia AA, Valdrighi L, Souza-Filho FJ (2003). Effectiveness of 2% chlorhexidine gel and calcium hydroxide against Enterococcus faecalis in bovine root dentine in vitro. Int Endod J.

[5] Gomes BPFA, Sato E, Ferraz CCR, Teixeira FB, Zaia AA, Souza-Filho FJ (2003). Evaluation of time required for recontamination of coronally sealed canals medicated with calcium hydroxide and chlorhexidine. Int Endod J.

[6] Shokraneh A, Farhad AR, Farhadi N, Saatchi M, Hasheminia SM (2014). Antibacterial effect of triantibiotic mixture versus calcium hydroxide in combination with active agents against Enterococcus faecalis biofilm. Dent Mater J.

[7] Guneser MB, Akbulut MB, Eldeniz AU (2013). Effect of various endodontic irrigants on the push-out bond strength of biodentine and conventional root perforation repair materials. J Endod.

[8] Bidar M, Naderinasab M, Talati A, Ghazvini K, Asgari S, Hadizadeh B, Gharechahi M, Mashadi NA (2012). The effects of different concentrations of chlorhexidine gluconate on the antimicrobial properties of mineral trioxide aggregate and calcium enrich mixture. Dent Res J (Isfahan).

[9] Mittag SG, Eissner C, Zabel L, Wrbas KT, Kielbassa AM (2012). The influence of chlorhexidine on the antibacterial effects of MTA. Quintessence Int.

[10] Arruda RA, Cunha RS, Miguita KB, Silveira CF, De Martin AS, Pinheiro SL, Rocha DG, Bueno CE (2012). Sealing ability of mineral trioxide aggregate (MTA) combined with distilled water, chlorhexidine, and doxycycline. J Oral Sci.

[11] Mohammadi Z, Abbott PV (2009). The properties and applications of chlorhexidine in endodontics. Int Endod J.

[12] Zehnder M (2006). Root canal irrigants. J Endod.

[13] Mohammadi Z, Shalavi S (2012). Is Chlorhexidine an Ideal Vehicle for Calcium Hydroxide? A Microbiologic Review. Iran Endod J.

[14] Rossi-Fedele G, Doğramacı EJ, Guastalli AR, Steier L, Poli de Figueiredo JA (2012). Antagonistic interactions between sodium hypochlorite, chlorhexidine, EDTA, and citric acid. J Endod.

[15] Gomes BP, Vianna ME, Zaia AA, Almeida JF, Souza-Filho FJ, Ferraz CC (2013). Chlorhexidine in endodontics. Braz Dent J.

[16] Prado M, Simao RA, Gomes BPFA (2013). Effect of different irrigation protocols on resin sealer bond strength to dentin. J Endod.

[17] Basrani BR, Manek S, Sodhi RNS, Fillery E, Manzur A (2007). Interaction between sodium hypochlorite and chlorhexidine gluconate. J Endod.

[18] Thomas JE, Sem DS (2010). An In Vitro Spectroscopic Analysis to Determine Whether Para-Chloroaniline Is Produced from Mixing Sodium Hypochlorite and Chlorhexidine. J Endod.

[19] Nowicki JB, Sem DS (2011). An in vitro spectroscopic analysis to determine the chemical composition of the precipitate formed by mixing sodium hypochlorite and chlorhexidine. J Endod.

[20] Marchesan MA, Pasternak Junior B, Afonso MM, Sousa-Neto MD, Paschoalato C (2007). Chemical analysis of the flocculate formed by the association of sodium hypochlorite and chlorhexidine. Oral Surg Oral Med Oral Pathol Oral Radiol Endod.

[21] Basrani BR, Manek S, Mathers D, Fillery E, Sodhi RN (2010). Determination of 4-chloroaniline and its derivatives formed in the interaction of sodium hypochlorite and chlorhexidine by using gas chromatography. J Endod.

[22] Krishnamurthy S, Sudhakaran S (2010). Evaluation and prevention of the precipitate formed on interaction between sodium hypochlorite and chlorhexidine. J Endod.

[23] Chhabra RS, Huff JE, Haseman JK, Elwell MR, Peters AC (1991). Carcinogenicity of p-chloroaniline in rats and mice. Food Chem Toxicol.

[24] Bui TB, Baumgartner JC, Mitchell JC (2008). Evaluation of the interaction between sodium hypochlorite and chlorhexidine gluconate and its effect on root dentin. J Endod.

[25] Valera MC, Chung A, Menezes MM, Fernandes CE, Carvalho CA, Camargo SE, Camargo CH (2010). Scanning electron microscope evaluation of chlorhexidine gel and liquid associated with sodium hypochlorite cleaning on the root canal walls. Oral Surg Oral Med Oral Pathol Oral Radiol Endod.

[26] Akisue E, Tomita VS, Gavini G, Poli de Figueiredo JA (2010). Effect of the combination of sodium hypochlorite and chlorhexidine on dentinal permeability and scanning electron microscopy precipitate observation. J Endod.

[27] Vivacqua-Gomes N, Ferraz CCR, Gomes BPFA, Zaia AA, Teixeira FB, Souza-Filho FJ (2002). Influence of irrigants on the coronal microleakage of laterally condensed gutta-percha root fillings. Int Endod J.

[28] Gonzalez-Lopez S, Camejo-Aguilar D, Sanchez-Sanchez P, Bolanos-Carmona V (2006). Effect of CHX on the decalcifying effect of 10% citric acid, 20% citric acid, or 17% EDTA. J Endod.

[29] Rasimick BJ, Nekich M, Hladek MM, Musikant BL, Deutsch AS (2008). Interaction between chlorhexidine digluconate and EDTA. J Endod.

[30] Athanassiadis B, Abbott PV, Walsh LJ (2007). The use of calcium hydroxide, antibiotics and biocides as antimicrobial medicaments in endodontics. Aust Dent J.

[31] Almyroudi A, Mackenzie D, McHugh S, Saunders WP (2002). The effectiveness of various disinfectants used as endodontic intracanal medications: an in vitro study. J Endod.

[32] Schafer E, Bossmann K (2005). Antimicrobial efficacy of chlorhexidine and two calcium hydroxide formulations against Enterococcus faecalis. J Endod.

[33] Haenni S, Schmidlin PR, Mueller B, Sener B, Zehnder M (2003). Chemical and antimicrobial properties of calcium hydroxide mixed with irrigating solutions. Int Endod J.

[34] Ercan E, Dalli M, Dulgergil CT (2006). In vitro assessment of the effectiveness of chlorhexidine gel and calcium hydroxide paste with chlorhexidine against Enterococcus faecalis and Candida albicans. Oral Surg Oral Med Oral Pathol Oral Radiol Endod.

[35] Önçag̈ Ö, Gogulu D, Uzel A (2006). Efficacy of various intracanal medicaments against Enterococcus faecalis in primary teeth: an in vivo study. J Clin Pediatr Dent.

[36] Lin YH, Mickel AK, Chogle S (2003). Effectiveness of selected materials against Enterococcus faecalis: part 3 The antibacterial effect of calcium hydroxide and chlorhexidine on Enterococcus faecalis. J Endod.

[37] Evans MD, Baumgartner JC, Khemaleelakul S, Xia T (2003). Efficacy of calcium hydroxide: chlorhexidine paste as an intracanal medication in bovine dentin. J Endod.

[38] Lindskog S, Piercs AM, Blomiöf L (1998). Ghlorhexidine as a root canal medicament for treating inflammatory lesions in the periodontal space. Dent Traumatol.

[39] Waltimo TMT, Ørstavik D, Siren EK, Haapasalo MPP (1999). In vitro susceptibility of Candida albicans to four disinfectants and their combinations. Int Endod J.

[40] Roberts HW, Toth JM, Berzins DW, Charlton DG (2008). Mineral trioxide aggregate material use in endodontic treatment: a review of the literature. Dent Mater.

[41] Stowe TJ, Sedgley CM, Stowe B, Fenno JC (2004). The effects of chlorhexidine gluconate (0.12%) on the antimicrobial properties of tooth-colored ProRoot mineral trioxide aggregate. J Endod.

[42] Holt DM, Watts JD, Beeson TJ, Kirkpatrick TC, Rutledge RE (2007). The anti-microbial effect against enterococcus faecalis and the compressive strength of two types of mineral trioxide aggregate mixed with sterile water or 2% chlorhexidine liquid. J Endod.

[43] Hernandez EP, Botero TM, Mantellini MG, McDonald NJ, Nor JE (2005). Effect of ProRoot MTA mixed with chlorhexidine on apoptosis and cell cycle of fibroblasts and macrophages in vitro. Int Endod J.

[44] Sumer M, Muglali M, Bodrumlu E, Guvenc T (2006). Reactions of connective tissue to amalgam, intermediate restorative material, mineral trioxide aggregate, and mineral trioxide aggregate mixed with chlorhexidine. J Endod.

[45] Yan P, Peng B, Fan B, Fan M, Bian Z (2006). The effects of sodium hypochlorite (525%), Chlorhexidine (2%), and Glyde File Prep on the bond strength of MTA-dentin. J Endod.

[46] Kogan P, He J, Glickman GN, Watanabe I (2006). The effects of various additives on setting properties of MTA. J Endod.

[47] Shahi S, Rahimi S, Yavari HR, Shakouie S, Nezafati S, Abdolrahimi M (2007). Sealing ability of white and gray mineral trioxide aggregate mixed with distilled water and 012% chlorhexidine gluconate when used as root-end filling materials. J Endod.

